# Comparative Analyses of Physicochemical Properties of Artisanal Refined Gasoline and Regular Automotive Gasoline

**DOI:** 10.3389/fchem.2020.00753

**Published:** 2020-10-02

**Authors:** Godwin J. Udo, Joachim J. Awaka-Ama, Emaime J. Uwanta, Ifiok O. Ekwere, Igwe R. Chibueze

**Affiliations:** ^1^Petroleum and Geochemistry Research Group, Department of Chemistry, Akwa Ibom State University, Uyo, Akwa Ibom State, Nigeria; ^2^NNPC Towers, Abuja, Nigeria

**Keywords:** physicochemical, gasoline, sulfur content, specific gravity, octane number

## Abstract

Physicochemical properties of artisanal refined gasoline (ARG) and regular automotive gasoline (RAG) sampled from the Eastern Obolo Creek and Mkpat Enin, Akwa Ibom State, Nigeria were investigated. This was to compare the physicochemical properties of the two gasoline samples with each other and their compliance with American Society for Testing and Materials (ASTM) standards. The finding revealed an antiknock index of RAG (91.15%) and ARG (83.05%), atmospheric distillation of RAG (185°C) and ARG (184°C), Reid vapor pressure of RAG (0.53 kg/cm^3^) and ARG (0.36 kg/cm^3^), gravity of RAG (0.771) and ARG (0.683), sulfur content of RAG (0.014%/wt) and ARG (0.02%/wt), while Flash point for RAG were Pensky Martens −25°C, Abel-Pensky −33°C and ARG Pensky Martens −27°C, Abel-Pensky −35.36°C, respectively. The research octane number, motor octane number, Reid vapor pressure, sulfur content, and specific gravity of RAG were (ASTM) compliant while only the final boiling point and sulfur content of ARG were within ASTM range. Based on the findings, the LRG might have been poorly refined or adulterated and could constitute problems in automotive engines if used. However, this crude technology can be upgraded and the gasoline quality improved through alkylation, isomerization, and cyclization. Artisanal refiners should be trained to become proficient with the intent of becoming incorporated into the upstream petroleum sector.

## Introduction

Gasoline is a complex mixture of hydrocarbons and other chemical compounds used as fuel for spark-ignition internal combustion engines, primarily in light duty transportation vehicles (David et al., 2018). Gasoline is in high demand in developing countries because of an increase in population, with a resultant increase in vehicular and industrial activities. Furthermore, refineries are producing at below installed capacities or are not functioning at all, which has resulted in the inability to refine enough gasoline to meet local consumption. Artisanal refining activity in the Niger Delta is increasing (Yabrade and Tanee, 2016). In artisanal refining, crude oil is boiled at atmospheric temperature; the resultant fumes are condensed and collected in tanks and used locally as automotive fuel. This local refining skill is believed to have been drawn from indigenous technology (Goodnews and Wordu, 2019). The artisanal refineries operating in the creeks of the Niger Delta, though illegal, provide employment to the locals as well as bridge the gaps in the availability and supply of refined petroleum products in the oil-bearing communities of the region (Brandes and Möller, 2008; Goodnews and Wordu, 2019; Addeh, 2020). NNPC in its report stated that Nigeria is not currently refining crude oil and therefore the corporation distributes only imported petroleum products in the country.

Though gasoline produced by artisan refiners is not tested well enough to certify its compliance to any local or international set parameters; it still cushions the effect of gasoline scarcity. Makeshift techniques are used by artisan refiners in processing the raw crude oil, *via* thermal cracking, into useful products. These procedures could be unsophisticated and not very safe, however it could be effective. The petroleum fractions obtained by local refiners are skeptically referred to as “bunkering oil” or adulterated products. Indigenous innovation and ingenuity in harnessing our natural resources should be appreciated, regulated, and the products assessed if they meet local and international specifications. Also, there is a need to assess the level of quality compliance of the gasoline samples distributed in the area to guard against environmental pollution and engine malfunctioning. According to Vempatapu and Kanaujia (2017), physicochemical properties like distillation profile, research octane number (RON), motor octane number (MON), and Reid vapor pressure are frequently used to detect the adulteration and quality of gasoline. It is on this basis that this research was designed to compare the physicochemical properties of regular automotive gasoline and locally refined gasoline and their compliance with ASTM standards.

## Materials and Methods

### Sample Collection

Five samples of artisanal refined gasoline (ARG) and regular automotive gasoline (RAG) were randomly collected from the Eastern Obolo Creek and Mkpat Enin, Akwa Ibom State, Niger Delta, Nigeria. Labeled amber sample bottles (2.5 L) with glass stoppers were used to collect the gasoline samples. At each sampling station, the sample bottle was rinsed with the gasoline sample to be collected. The sample was introduced into the sample bottle *via* the dispenser nozzle, labeled, and transported to the laboratory for treatment and analysis. ASTM standards were used as reference standards and all samples were analyzed according to ASTM test methods.

### Determination of Research Octane Number (RON) and Motor Octane Number (MON)

The gasoline samples, 300 mL each, were injected into the carburetor, the knock meter turned on, and the selector valve allowed to run for some minutes to attain equilibrium. The cylinder height of the knock meter reading was adjusted between 45 and 47 and finally to 50 after locating the fuel level for maximum knocks. The research octane number gave the maximum knock fuel ratio and standard knock intensity. The research octane number was determined at a low speed of 600 rpm while the motor octane number was determined at a higher speed of 900 rpm. This was conducted according to the ASTM-D2699 standard procedure using knock meter model ZX101C. The research octane number was obtained by varying the comparison ratio of the evaluated gasoline sample until knocking was observed in a knock meter. A mixture of iso-octane and n-heptane (94.20:5.8) was used to run the engine until the engine knocked again. Then the RON of the evaluated gasoline was 94.20.

Antiknock was calculated using:

$$\begin{array}{l} \text{Antiknock index }\left(\text{AKI}\right)=\frac{R+M}{2} \end{array}$$

*where*, R = Research Octane Number, M = Motor Octane Number (Nadkarnim Kishore R. A., 2000).

### Determination of Atmospheric Pressure Distillation

Determination of the initial and final boiling point of the gasoline samples were conducted using atmospheric pressure distillation unit model 11860-3U according to the ASTM-D86 standard test method (ASTM, 2006b). The gasoline sample (100 mL) was added into a round bottom flask containing anti-bumping granules. The distillation machine was switched on and the temperature was adjusted to 300°C. The initial boiling point (IBP) temperature of the gasoline samples were recorded immediately when the first drop of gasoline entered the measuring cylinder. The temperature of the distillation machine was increased to take the final boiling point (FBP) reading. Also, the total recovery (TR) temperature was recorded.

### Reid Vapor Pressures (RVP) Analysis

The Reid vapor pressure analysis of the samples was carried out according to the ASTM test method D323 using the Reid vapor pressure analyzer (P-700-1.00 model). The gasoline sample (50 mL) was introduced into the Reid vapor pressure machine and submerged into the Reid vapor pressure water bath. The temperature was adjusted to 38°C. After 30 min, the light fraction of the gasoline sample was vaporized and the pressure of the escaping vapor was recorded.

### Determination of Specific Gravity (S.G) and API Gravity

A measuring cylinder (100 ml) was swirled with a small portion of the test sample, blown dry, and 50 ml of the test sample added. A hydrometer, calibrated from 0.50 to 0.85, was immersed into the sample and the specific gravity (S.G) was recorded. Also, a thermometer was inserted into the measuring cylinder and the final temperature of the sample was recorded and corrected to °F. This was done according to the ASTM test method D1298 (ASTM, 2006a). The API gravity was calculated with this formula:

$$\begin{array}{l} \text{APIGravity}=\frac{141.5}{S.G}-131.5 \end{array}$$

### Determination of Sulfur Content

Sulfur content was determined using the Raney Nickel method. This method is efficient and gives reliable results (Hendsbee et al., 2006; Nejad and Miran Beigi, 2015) when a sulfur analyzer or the ICP–GC MS equipment is not readily available. A nickel aluminum catalyst of 0.6 g was weighed into a distillation flask. Ten mL of NaOH (2.5 N) was added, after which the mixture was allowed to completely react (vigorous reaction). The reacting mixture was covered with aluminum foil and left overnight. Another nickel aluminum catalyst was added to the distillation flask, washed three times with distilled water, and rinsed with 10 mL IPA to remove all water samples. A weighed amount of the sample (100 mL) was added to the flask washed down with 10 mL of IPA. The flask was placed on a heater and heated. Fifteen mL each of acetone and NaOH (1 N) were added to the scrubber (Common Sulfur Test Methods, 2007). Nitrogen gas was passed through the sample and allowed to desulphurize for 20 min. Three drops of dithizone was added to the scrubber, containing acetone and 1 N NaOH, to obtain a pink coloration. Ten mL of HCl (1 N) was introduced into the flask through a funnel, followed by the addition of mercuric acetate until there was no color change. The final reading of the mercuric acetate was taken (ASTM, 2008).

$$\begin{array}{l} \text{\%wt sulfur}=\frac{A\times T}{W} \end{array}$$

*where A* = net volume of mercuric acetate, T = titer value of mercuric acetate, W = weight of sample in grams.

### Determination of Flash Point

The flash points for the samples were determined using a Flash point tester (closed model K16270) for the upper temperature and a Pour/Cloud point tester (model PP-F3B4) for the lower temperature, according to ASTM-D86 standard procedure. The gasoline sample (100 mL) was introduced into a round bottom flask containing anti-bumping pellets. The distillation machine was switched on and the temperature was adjusted to 300°C. The initial boiling point (IBP) temperature was recorded immediately when the first drop of the sample entered the measuring cylinder. The 10% ASTM D86 temperatures were determined and the flash points were calculated using the equation given below.

$$\begin{array}{l} \frac{\text{1}}{\text{TFP}}=-\text{0}.\text{014568}-\frac{\text{2}.\text{8497}}{\text{T1}}-\text{1}.\text{903}\times {\text{10}}^{-3}lnT_{1} \end{array}$$

*where*, T_FP_ = Flash point (Penskey-Martens closed cup ASTM D93) of the gasoline.

T_1_ = ASTM 10% temperature for the gasoline or normal boiling point.

It should be noted that, the flash point and the ASTM 10% temperature for the gasoline are measured in degrees Rankine. 1R = 196.7F.

## Results and Discussion

### Research Octane Number, Motor Octane Number, and Antiknock Index (AKI)

The average research octane number (RON) of RAG was 94.20% within the ASTM standard range (ASTM D2700-19, 2019) (Table 1). Conversely the average research octane number of ARG was 82.9% lower than the ASTM minimum limit (Table 2), implying that artisanal refined gasoline can trigger engine knock. The low RON of ARG may be as a result of poor refining operational conditions. The research octane number and motor octane number are used to measure the octane rating of gasoline. RON measures the ability of gasoline to knock or ping in an engine. Knocking is the metallic noise produced in a spark ignited engine when low octane rating gasoline is used (Onyinye and Okoye, 2015). Octane rating of gasoline determines the tendency of gasoline to resist pre-ignition during compression in an engine cylinder (David et al., 2018). An antiknock agent is an additive in gasoline that reduces engine knocking by increasing the octane rating of the fuel. This occurs by raising the temperature and pressure of auto-ignition (Sheet, 2011). The antiknock index (ANI) of RAG and ARG were 91.15 and 83.05, respectively (Table 2). The antiknock index is the average of RON and MON. The USA requires an antiknock index of 91 AKI or higher for premium gasoline and 87 AKI for regular gasoline cylinders (David et al., 2018). A higher AKI gasoline results in higher vehicle fuel economy and better performance. The volatility and octane quality of gasoline are of the utmost importance in determining the quality of gasoline (Chikwe et al., 2016). Straight chain alkanes have more knocking tendency compared to branched chain alkanes. Antiknocking agents such as tetraethyl lead, act as free radical chain inhibitors and therefore check the propagation of the explosive chain thus reducing knocking.

**Table 1:** Research octane number for regular automotive gasoline (RAG) and artisanal refine gasoline (ARG).

**Sample location**	**Sample Volume (mL)**	**RON%(RAG)**	**RON %(ARG)**	**ASTM Standard**
SL1	300	92.80	94.20	91.00
SL2	300	94.20	86.50	
SL3	300	96.20	83.08	
SL4	300	93.60	80.28	
SL5	300	94.20	83.79	
$\frac{\sum x}{f}$		**94.20**	**82.90**	**91.00**

**Table 2:** Motor octane number for regular automotive gasoline (RAG) and artisanal refine gasoline (ARG).

**Sample location**	**Sample Volume (mL)**	**MON%RAG**	**MON % -ARG**	**ASTM Standard (%)**
SL1	50	92.00	84	
SL2	50	89.00	86	
SL3	50	86.50	82	
SL4	50	86.00	81	
SL5	50	87.00	83	
$\frac{\sum x}{f}$		**88.10**	**83.20**	**83.00**
Antiknock index		**91.15**	**83.05**	**87.00**

*Bold values indicate the average*.

### Atmospheric Pressure Distillation Profile

The initial boiling point of RAG was 48°C, which was higher than the ASTM range of 35°–39°C while the final boiling point of RAG was 185°C, lower than the ASTM range of 195°–204°C (ASTM, 2006b) (Table 3). The initial boiling point of ARG was 38°C and was within the ASTM range of 35°–39°C. Also, the final boiling point of ARG was 184°C below the ASTM range of 195–102°C (Table 3). In a related study conducted by (Onyinye and Okoye, 2015), 39 ± 0.817 and 204 ± 0.817°C were recorded for initial and final boiling points. The temperature range over which the gasoline mixture boils is known as the distillation profile (David et al., 2018). The American Standard of Testing and Materials (ASTM) for gasoline requires that 10 mL recovery should not exceed 60°C (Table 3). The 10 mL recovery boiling point is the range at which a spark plug will first ignite (Onojake et al., 2012). Similarly according to the ASTM reference limit, the 50 mL recovery and final boiling point should not exceed 110° and 195°–204°C, respectively (Table 3). When compared with the ASTM reference limit, the 10 mL recovery temperature of RAG was 71°C greater than the recommended 60°C (Table 3). The 10 mL recovery temperature of ARG was 51°C within the reference limit. Also, the 50 mL recovery temperatures for RAG and LRG were 100° and 84°C, respectively, and were within the ASTM 110°C limit (Table 3). The investigation also revealed that the final boiling points of RAG and ARG were 185° and 184°C, respectively, and were within ASTM acceptable limits (195°–204°C). In regard to atmospheric pressure distillation, RAG was slightly adulterated compared to ARG. This is because the 10 ml recovery temperature 71°C (Table 3), exceeded the ASTM limit of 60°C. RAG might have been poorly refined, adulterated, or poorly blended (David et al., 2018).

**Table 3:** Atmospheric pressure distillation.

**% Recovery**	**Temperature of Recovery (TR) (**^****°****^**C)**		
	**RAG**	**ARG**	**ASTM Standard**
Initial boiling point (IBP)	48	38	35–39
5	64	42	
10	71	51	60
20	78	58	
30	85	64	
40	92	72	
50	100	84	110
60	107	92	
70	115	115	
80	126	130	
90	144	163	170
Final Boiling point (FBP)	185	184	195–204
TR (%)	99%	99%	

### Reid Vapor Pressure (RVP)

The Reid vapor pressure of RAG was 0.53 kg/cm^2^ within the permissible range of 0.45–0.60 as recommended by the American Standard of Testing and Material (Table 4). Reid vapor pressure (RVP) measures vapor pressure of a gasoline blend at 100 degrees Fahrenheit (°F) (David et al., 2018). It is a measure of the volatility of the gasoline when in use in automotive engines. A high RVP of gasoline blend makes some components of the gasoline volatilize when exposed to the atmosphere (David et al., 2018). Conversely, the RVP of LRG was 0.36 kg/cm^2^ below the minimum ASTM limit of 0.45 kg/cm^2^ (Table 4) (Chilingar et al., 2005). Peretomode (2018), in a similar work reported a RVP of 0.37–0.41 psi. The low RVP of ARG samples implies that if used in an automotive engine, starting the engine at low temperatures may be a problem (David et al., 2018). It also suggests that the sample contained a heavy hydrocarbon fraction which could result from poor refinery operation conditions, or adulteration (Onojake et al., 2013). In hotter climates, gasoline components of higher molecular weight and thus lower RVP are used, very high RVP results in “vapor lock.” Very low RVP in cold climates causes the failure of car engines to start while in hot climates, excessive volatility results in what is known as “vapor lock.”

**Table 4:** Reid vapor pressure (RVP) (kg/cm^2^) at 37.8°C for RAG and LRG.

**Sample volume (mL)**	**Reid vapor pressure RAG**	**Reid vapor pressure ARG**	**ASTM Standard (ASTM, 2006b)**.
50	0.57	0.38	
50	0.51	0.36	
50	0.56	0.38	
50	0.52	0.31	
50	0.49	0.37	
Average (RVP)	**0.53**	**0.36**	0.45–0.60

*Bold values indicate the average*.

### Specific Gravity

The result of the specific gravity of regular automotive gasoline and locally refined gasoline samples are presented in Table 5. The specific gravity of regular automotive gasoline was 0.7708 within the ASTM range of 0.75–0.85. In regard to the specific gravity, the RAG samples will not constitute a problem to users. Conversely, the result of the specific gravity of the locally refined gasoline was 0.6832 which was lower than the minimum ASTM standard. The values are within the ASTM acceptable range of 0.75–0.85; this can cause damage if used in an automotive engine. Gasoline samples with a specific gravity >0.75 will have a high burning rate and thus would be required in larger quantities. It might also result in complete detonation (pinging) of the spark-ignited engine (Alang et al., 2018).

**Table 5:** Specific gravity of RAG and ARG.

**Sample volume (mL)**	**Specific gravity RAG**	**Specific gravity ARG**	**ASTM Standard**
50	0.7898	0.7515	
50	0.7701	0.6635	
50	0.7852	0.6920	
50	0.7980	0.6985	
50	0.7109	0.6105	
Average	**0.7708**	**0.6832**	**0.75–0.85**

*Bold values indicate the average*.

### Flash Point

The results of the flash point Pensky Martens and Abel-Pensky for RAG were −25° and −33°C, respectively (Table 6). Similarly, the flash point Pensky (Martens and Abel-Pensky) for ARG were −27° and −35.36°C, respectively. The flash points of the RAG and ARG were higher than the ASTM standard of −43°C (ASTM, 2007). This implies that the gasoline samples under investigation could easily ignite because the higher the flash point the easier the rate of ignition and explosion (Brandes and Möller, 2008). This also means that the gasoline samples under study could pose a risk during storage, handling, and transportation (Abdelkhalik et al., 2018). A flash point is the lowest temperature of a liquid, at which the vapor of the sample is ignited in the presence of an ignition source and the flame propagates across the surface of the sample (Brandes and Möller, 2008; Abdelkhalik et al., 2018). Flash point is important in the safe handling, storage, and transportation of gasoline.

**Table 6:** Flash point (Pensky Martens ^o^C, Abel-Pensky closed cup ^o^C) of RAG and ARG.

**Sample volume (mL)**	**Flash Point RAG**		**Flash Point ARG**	
	**Pensky Martens (°C)**	**Abel-Pensky closed cup (°C)**	**Pensky Martens (°C)**	**Abel-Pensky closed cup (°C)**
50	−26	−34	−25	−36
50	−24	−32	−28	−37
50	−25	−33	−27	−35.8
50	−24	−32	−26	−33
50	−26	−34	−29	−35
Average flash point	−**25**	−**33**	−**27**	−**35.36**

*Bold values indicate the average*.

### Sulfur Content

The sulfur content of regular automotive gasoline and locally refined gasoline samples under investigation were 0.0143 and 0.0238%, respectively (Table 7). Comparatively, the sulfur content of the locally refined gasoline samples were higher than the regular automotive gasoline samples (ASTM D452-18a, 2018). The values of sulfur content of the two gasoline samples were within the ASTM acceptable range of 0.0100–0.0500%. European countries permit up to 0.001% wt of sulfur in their gasoline (Assi, 2008). Elemental sulfur levels as low as 2–3 μg/g may be sufficient to induce corrosion of the silver alloy level-sensor elements in fuel tanks. Also, exhaust emissions from vehicles are prominent sources of harmful pollutants, like sulfur (IV) oxide (SO_2_), formed from combustion from sulfur-containing compounds in gasoline. Sulfur (lV) oxide is an acidic gas associated with health hazards when inhaled. Automobile gasoline with low percentages of sulfur are recommended because of environmental concerns. Elemental sulfur appears to be present in gasoline primarily in the form of S8 rings with smaller amounts of S6 and S7 (Pauls, 2010).

**Table 7:** Sulfur content of regular automotive gasoline (RAG) and artisanal refined gasoline (ARG).

**Sample volume (mL)**	**Sulfur Content (% wt) RAG**	**Sulfur content (% wt) ARG**	**ASTM Standard**
100	0.0159	0.0277	(ASTM D975-07, 2007)
100	0.0111	0.0241	
100	0.0118	0.0229	
100	0.0126	0.0245	
100	0.0201	0.0198	
$(Average=\frac{\sum x}{f}$)	**0.0143**	**0.0238**	**0.0100–0.0500**

The comparative physicochemical properties of RAG and ARG are summarized in Table 8. The results indicate the values of the different physicochemical properties of regular automotive gasoline and artisanal refined gasoline compared to the ASTM standards. A way to increase or decrease the RAG and ARG values so that they comply with ASTM and other international standards is by strictly adhering to standard petroleum refining processes. Non-compliance with standard refining processes may be responsible for the deviation in some of the values.

**Table 8:** Summary of comparative physicochemical properties of RAG and ARG.

**Physicochemical Properties**	**RAG**	**ARG**	**ASTM Standards**
Research octane number	94.20	82.90	91.00 (ASTM D2700-19, 2019)
Motor octane number	88.10	83.20	83.00 (ASTM D2700-19, 2019)
Atmospheric pressure distillation	185	184	195-204 (ASTM, 2006b)
Reid vapor pressure	0.53 kg/cm^2^	0.36 kg/cm^2^	0.45–0.60 kg/cm^2^ (ASTM, 2006b).
Specific gravity	0.7708 g cm−3	0.6832 g cm−3	0.75–0.85 gcm^−3^ (ASTM D452-18a, 2018)
Flash point	−33^o^C	−35.36^o^C	−35.36^o^C (ASTM, 2007)
Sulfur content	0.0143%wt	0.0238%wt	0.0100–0.0500%wt (ASTM D975-07, 2007)

## Conclusion

The results of this study revealed that the research octane number, motor octane number, Reid vapor pressure, sulfur content, and specific gravity of regular automotive gasoline were within ASTM specifications while initial boiling point and flash point deviated from the ASTM range. In artisanal refined gasoline only atmospheric distillation and sulfur contents were within ASTM acceptable standards. The research octane number, motor octane number, Reid vapor pressure, and specific gravity of locally refined gasoline deviated from the ASTM standards. Based on these findings, the artisanal refined gasoline might have been poorly refined or adulterated and could constitute problems in automotive engines when used. The quality of the artisanal gasoline can be enhanced through upgrading refinery operation conditions and the introduction of gasoline additives. Nigeria needs to develop local technology in order to attain self-sufficiency in the petroleum sector. The federal government should legislate a legal framework to regulate the activities of artisanal refineries. This will ensure environmental protection, create confidence among the refiners, promote local technology, and generate employment and economic sustainability.

## Data Availability Statement

The raw data supporting the conclusions of this article will be made available by the authors, without undue reservation.

## Author Contributions

All authors listed have made a substantial, direct and intellectual contribution to the work, and approved it for publication.

## Conflict of Interest

IC was employed by the company, Nigerian National Petroleum Corporation. The remaining authors declare that the research was conducted in the absence of any commercial or financial relationships that could be construed as a potential conflict of interest.
